# Chest radiography patterns of COVID-19 pneumonia in Kumasi, Ghana

**DOI:** 10.4314/gmj.v54i4.9

**Published:** 2020-12

**Authors:** Augustina Badu-Peprah, Ijeoma Anyitey-Kokor, Allswell Ackon, Obed K Otoo, Nana A K Asamoah, Theophilus K Adu-Bredu

**Affiliations:** 1 Radiology Directorate - Komfo Anokye Teaching Hospital, Kumasi, Ghana; 2 Radiology Department, School of Medicine and Dentistry, Kwame Nkrumah University of Science and Technology, Kumasi, Ghana; 3 Obstetrics and Gynaecology Directorate - Komfo Anokye Teaching Hospital, Kumasi, Ghana

**Keywords:** COVID-19, chest radiography, ground-glass opacities, consolidation, symptoms

## Abstract

**Objectives:**

To document the pattern of chest radiographic findings in coronavirus disease 2019 (COVID-19) patients with moderate to severe disease.

**Design:**

Retrospective cross-sectional study.

**Setting:**

The study site was Komfo Anokye Teaching Hospital (KATH) located at Bantama in Kumasi, the capital town of the Ashanti Region. It is the second largest hospital in the country and the major referral site for Ashanti region and the northern part of the country. The hospital hosts a highly infectious isolation unit (HIIU) for COVID-19 patients with moderate to severe infections and receives referred cases from the region and within the hospital.

**Participants:**

The study involved 27 patients admitted to the HIIU at KATH with COVID-19 infection who underwent chest X-ray as part of their investigations.

**Results:**

The study enrolled 12 males and 15 females. The common comorbidities were hypertension and diabetes. Chest x-ray findings in most of the patients (81.5%) revealed ground-glass opacities while a few of them (18.5%) had lung consolidations without ground-glass opacities. For those with ground-glass opacities, eight (29.6%) had superimposed consolidation. All the participants had positive chest x-ray findings.

**Conclusion:**

The chest x-ray findings in the 27 COVID-19 positive patients with moderate to severe disease on admission at the KATH HIIU enrolled in this study showed significant pulmonary abnormalities. The predominant pulmonary abnormalities were bilateral peripheral ground-glass opacities with the lower lung zones being mostly affected.

**Funding:**

Funding from the Radiology Directorate, Komfo Anokye Teaching Hospital, Kumasi, Ghana

## Introduction

In December 2019, the coronavirus disease 2019 (COVID-19) outbreak was reported in Wuhan, China, and quickly spread to other regions in China,^1^ as well as other countries across the world in the last few months. The outbreak was declared a Public Health Emergency of International Concern (pandemic) on 11^th^ March 2020 by the World Health Organization (WHO).^2^ The rate of COVID-19 infection is currently on the increase in Ghana with over 40,000 confirmed cases and over 215 COVID-19-related deaths according to the latest data by Ghana Health Service (as at 11^th^ July 2020). 3 Considering the fact that the Ghana Health Service is now doing targeted testing in Ghana, the true number of cases may exceed the reported numbers.

This novel disease is enigmatic to scientists and clinicians across the world since it has varied outcomes irrespective of the age, race, gender, and geographic location. The presentation of positive COVID-19 patients varies from asymptomatic manifestation to patients requiring ventilators, severe neurological impairment, and death.^4^

During the management of the previous outbreak of severe acute respiratory syndrome (SARS) and middle east respiratory syndrome coronavirus (MERS-CoV), chest radiographs played a key role in the diagnosis and follow up of cases.

However, literature on COVID-19 so far has mainly focused on the use of computed tomography (CT) in identifying and monitoring disease progression.^1^,^5^ The use of CT in COVID-19 management is faced with several challenges. Mandatory decontamination of the CT room after scanning a COVID-19 patient disrupts the workflow in the radiology department of institutions which do not have a dedicated CT scan machine for imaging COVID-19 patients. Secondly, most hospitals in resource poor nations like Ghana do not have CT scan.

The chest radiograph even though less sensitive compared to CT in identifying features of early disease in the chest^6^ is a suitable alternative especially in hospitals within low to middle-income countries. This is supported by the high patient load and the long turnaround time for reverse transcription polymerase reaction (RT-PCR).^7^

Currently, most of the publications on the radiology of COVID-19 are mainly from Asia and Europe with little published from Sub-Saharan Africa. In hospitalized patients, routine chest radiographs are performed to assess the extent of lung changes and monitor lung response to treatment. In view of this, it is necessary to understand the radiographic appearance of lung changes in patients with moderate to severe symptoms in our population and determine the diagnostic value of chest × -rays (CXR).

The main objective of the study was to document the pattern of COVID-19 pneumonia on chest radiographs. We also sought to determine the sensitivity of chest x-rays in identifying pulmonary abnormalities due to moderate to severe COVID-19 pneumonia, to characterize the specific pulmonary abnormalities seen in the chest x-rays of patients with moderate to severe COVID-19 pneumonia and determine the zones of the lung that are predominantly affected.

## Methods

This is a retrospective cross-sectional study conducted at Komfo Anokye Teaching Hospital (KATH) located in Kumasi, Ghana. The hospital hosts a highly infectious isolation unit (HIIU) which receives COVID-19 patients with moderate to severe disease within the hospital and other facilities in the region. A dedicated mobile X-ray machine has been assigned to the unit. This study was conducted using the medical records and images from patients admitted to the HIIU.

### Inclusion criteria

Patients admitted at the HIIU with at least a chest radiograph and complete medical records.

### Exclusion criteria

HIIU patients with incomplete medical records or without a chest radiograph

### Data collection

Data used for the study was accessed from the medical records of the HIIU from 1st June to 9th July 2020. Patient identity was anonymised and replaced with autogenerated identification numbers. A predesigned questionnaire was used to extract the relevant information from the available patient records. Information documented included age, clinical symptoms, duration of symptoms, comorbidities and patient outcomes. Four experienced radiologists independently reported on each of the radiographs using an agreed and predetermined structured template. The Principal Investigator resolved any interobserver disparities.

### Image interpretation

The radiographic images were interpreted using the Fleischner Society glossary of terms for thoracic imaging.^8^ Ground-glass opacity was defined as an area of extensive hazy increased lung opacity with impaired visualization of margins of the pulmonary vessels. Consolidation was defined as a homogenous increase in attenuation of the lung parenchyma which obscures the margins of the airway walls and vessels. The findings on the chest radiographs were documented using a predetermined structured template.

### Data analysis

After compiling and tabulating the data, STATA version 16 statistical software was used to analyse the data.

### Ethical Issues

Ethical clearance was sought from the KATH Institutional Review Board and approval was given with reference number KATH-IRB/AP/075/20. Confidentiality of patients' information was maintained by assigning unique identifiers to each individual, which consequently was referenced in data capture and management.

## Results

Forty -one patients were admitted to HIIU over the study period and 27 (65.85%) met the inclusion criteria, made up of 12 males (44.4%) and 15 females (55.6%). Table 1 shows the age distribution of the patients.

**Table 1:** The age distribution of the enrolled patients

Age (years)	N (%)
**15–24**	1(3.7)
**25–34**	3(11.1)
**35–44**	2(7.4)
**45–54**	6(22.2)
**55–64**	7(25.9)
**65–74**	2(7.4)
**75–84**	4(14.8)
**>85**	2(7.4)
**Total**	27(100.0)

The three most predominant symptoms presented were fever 21(77.8%), cough 18(66.7%) and breathlessness 12(44.4%) as shown on *Table 2.0*. Most of the patients 19(70.37%) presented after 72 hours, two patients (7.4%) presented within 24 hrs, one patient (3.7%) between 24 and 48 hours and five patients (18.5%) between 48 and 72 hours.

**Table 2:** Clinical presentation of the enrolled COVID-19 patients

Symptoms (N=27)	N (%)
**Fever**	21(77.8)
**Cough**	18(66.7)
**Breathlessness**	12(44.4)
**Body weakness**	9(33.3)
**Dizziness**	6(22.2)
**Headache**	5(18.5)
**Palpitation**	5(18.5)
**Abdominal pain**	4(14.8)
**Dyspnoea**	4(14.8)
**Easy fatigue**	4(14.8)
**Vomiting**	4(14.8)
**Chest pain**	3(11.1)
**Chills**	3(11.1)
**Diarrhoea**	3(11.1)
**Constipation**	1(3.7)
**Dark urine**	1(3.7)
**Dysphagia**	1(3.7)
**General body pain**	1(3.7)
**Loss of appetite**	1(3.7)
**Loss of smell**	1(3.7)
**Nausea**	1(3.7)
**Odynophagia**	1(3.7)
**Sore throat**	1(3.7)
**Yellowish Sputum**	1(3.7)

Seventeen patients (63%) had two or more co-morbidities with 13(48.1%) having diabetes and hypertension as shown in Table 3.

**Table 3:** Presentation of co-morbidities in the enrolled COVID-19 patients

Co-morbidities	N (%)
**Hypertension only**	2(7.4)
**Diabetes only**	1(3.7)
**Sickle cell disease only**	3(11.1)
**Retroviral disease (AIDS) only**	2(7.4)
**Hypertension and Diabetes**	11(40.8)
**Hypertension, diabetes and sickle cell disease**	2(7.4)
Hypertension, diabetes and CKD*	1(3.7)
Hypertension and CKD*	1(3.7)
Diabetes and CKD*	1(3.7)
**Diabetes and Asthma**	1(3.7)
**None**	2(7.4)
**Total**	**27**

* CKD- Chronic Kidney Disease

Each of the 27 patients (100%) had significant imaging findings. Most of the patients 26(96%) had bilateral lung disease. The disease had a predominantly peripheral distribution in 21 patients (77.8%) and central in 3 patients (11.1%). Four patients (14.8%) had diffuse disease.

Zonal distribution of the disease was in two or more zones in 22(81.5%). Twenty- six (96.3%) of the radiographs had significant imaging findings in the lower zones. Middle zone disease was noted in 22 radiographs (81.5%) while upper zone disease was noted in 15 radiographs (55.6%).

**Figure 1:**
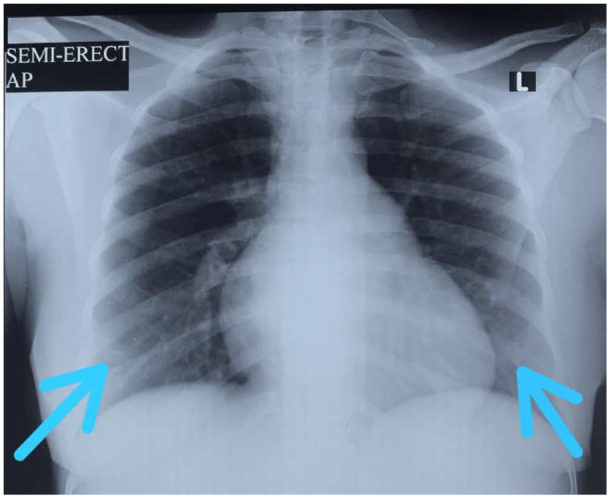
Bilateral symmetric ground- glass opacities in the lower lung zones in a teenage female who presented with a 6-day history of fever, cough, headache and easy fatiguability.

**Figure 2:**
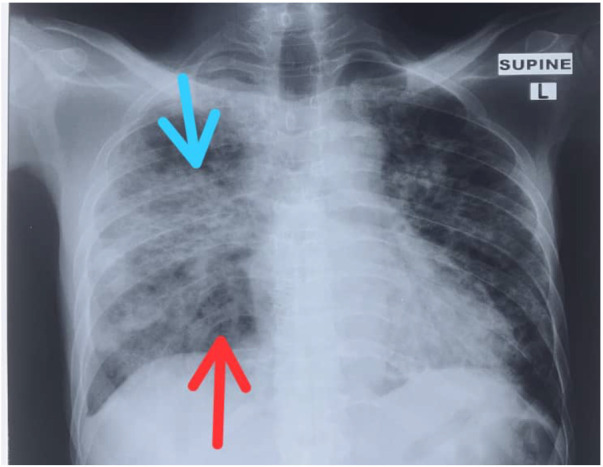
Bilateral asymmetric diffuse ground-glass opacities (red arrow) with superimposed consolidations (blue arrow) in a 53-year-old male who came in with a 4-day history of fever, cough, breathlessness, and dysphagia

The most predominant chest x-ray finding was groundglass opacities (Figure 1) seen in 22 (81.5%) patients. Five (18.5%) had consolidations without ground-glass opacities (Table 4). For those with ground-glass opacities, eight (29.6%) had superimposed consolidation (Figure 2) with one (3.7%) having bronchiolar dilatation.

**Table 4:** Radiological pattern of lung disease

Radiological Features	N (%)
**Consolidation**	2(7.4)
**Consolidation, Linear opacities, Architectural distortion**	1(3.7)
**Consolidation, Linear opacities, cardiomegaly**	1(3.7)
**Consolidation, Pleural effusion, Atelectasis**	1(3.7)
**Ground glass**	13(48.2)
**Ground glass, Bronchiolar dilation**	1(3.7)
**Ground glass, Consolidation**	8(26.6)
**Total**	27(100)

Most of the patients 20 (74.1%) had their RT-PCR test results after 24 hours with only 7 patients (25.9%) having their results in less than 24 hours. Only one patient was discharged during the study period. The patient, a 15-year-old sickle cell patient had symptoms like fever, cough, dark-coloured urine, and vomiting. He was discharged after 12 days following clinical improvement and complete resolution of the radiographic findings.

## Discussion

The top four symptoms reported by the patients enrolled in this study were fever, cough, breathlessness and body weakness consistent with published data by the World Health Organisation (WHO).^9^ Fifty-five percent of the patients were above 55 years with most of the patients being between 45–64 years. This age group has been found in other studies to be most susceptible to the infection.^10^ Only one patient had a history of travel from a COVID-19 endemic country to Ghana suggesting an extensive community spread of the disease.^11^

Our study revealed that all COVID-19 patients with moderate to severe disease had pulmonary abnormalities on chest x-ray in contrast to findings reported by similar studies but with different study population, as both severe and non-severe cases were used.^7^,^12^,^13^ The late presentation might have been a contributing factor, as 70.37% of the patients presented more than 3 days after onset of clinical symptoms. This finding also agrees with the statement by Wong et al^7^ that the sensitivity of chest xray increases with disease progression.

Ninety-three percent of the patient had comorbidities. However, even though the presence of comorbid conditions is said to be associated with moderate to severe disease, this study however could not establish that association.

Ground-glass opacity was observed in 22 (81.5%) of the patients and was the most common imaging finding followed by consolidation in 14(51.9% )of the patients. This is consistent with what was found by a systematic review of four COVID-19 studies by Ng et al.^14^ Cozzi et al^12^ have also documented similar findings with 62.8% ground-glass opacification and 57.7% consolidation. Wong et al^7^ on the other hand found more radiographs showing consolidation than ground glass opacity.

All the radiographs except one showed bilateral disease. This is supported by a meta-analysis of 19 studies conducted by Rodriguez – Morales et al which found bilateral pulmonary involvement in about 72% (95% CI 58.6 – 87.1) of COVID – 19 patients.^15^ The pulmonary changes were in the peripheral zones on most of the radiographs. Similar findings have been reported by Ng et al^14^ and Cozzi et al^12^. Wong et al^7^ has also reported that most of the findings were in the lower zones as was the case in this study which found lower zone involvement in all of the radiographs except one.

Due to the short duration of the study, the sample size was small which increased the likelihood of Type II error and hence decreased the statistical power of our study.

## Conclusion

The chest x-ray findings in the 27 COVID-19 positive patients with moderate to severe disease on admission at the KATH HIIU enrolled in this study showed significant pulmonary abnormalities. The predominant COVID-19 pulmonary abnormalities were bilateral peripheral ground-glass opacities with the lower lung zones being mostly affected.
